# Characterization of the DNA binding activity of structural protein VP1 from chicken anaemia virus

**DOI:** 10.1186/s12917-018-1465-5

**Published:** 2018-05-04

**Authors:** Guan-Hua Lai, Ming-Kuem Lin, Yi-Yang Lien, Jai-Hong Cheng, Fang-Chun Sun, Meng-Shiunn Lee, Hsi-Jien Chen, Meng-Shiou Lee

**Affiliations:** 1 Graduate Institute of Biotechnology, National Chung Hsing University, Taichung, 40402 Taiwan; 2 Department of Chinese Pharmaceutical Science and Chinese Medicine Resources, China Medical University, 91, Hsueh-Shih Road, Taichung, Taiwan; 3 Department of Veterinary Medicine, National Pingtung University of Science and Technology, Pingtung, 91201 Taiwan; 4 Center for Shockwave Medicine and Tissue Engineering, Department of Medical Research, Kaohsiung Chang Gung Memorial Hospital and Chang Gung University College of Medicine, Kaohsiung, 83301 Taiwan; 5 Department of Bioresources, Da-Yeh University, Changhua, 51591 Taiwan; 6 Research Assistance Center, Show Chwan Memorial Hospital, Changhua, 500 Taiwan; 7 Department of Safety, Health and Environmental Engineering, Ming Chi University of Technology, New Taipei, 24301 Taiwan

**Keywords:** Chicken anaemia virus, Capsid protein, VP1, DNA binding

## Abstract

**Background:**

Chicken anaemia virus (CAV) is commonly found in poultry. VP1 is the sole structural protein of CAV, which is the major component responsible for capsid assembly. The CAV virion consists of the VP1 protein and a viral genome. However, there is currently no information on the protein-nucleic acid interactions between VP1 and DNA molecules.

**Results:**

In this study, the recombinant VP1 protein of CAV was expressed and purified to characterize its DNA binding activity. When VP1 protein was incubated with a DNA molecule, the DNA molecule exhibited retarded migration on an agarose gel. Regardless of whether the sequence of the viral genome was involved in the DNA molecule, DNA retardation was not significantly influenced. This outcome indicated VP1 is a DNA binding protein with no sequence specificity. Various DNA molecules with different conformations, such as circular dsDNA, linear dsDNA, linear ssDNA and circular ssDNA, interacted with VP1 proteins according to the results of a DNA retardation assay. Further quantification of the amount of VP1 protein required for DNA binding, the circular ssDNA demonstrated a high affinity for the VP1 protein. The preferences arranged in the order of affinity for the VP1 protein with DNA are circular ssDNA, linear ssDNA, supercoiled circular dsDNA, open circular DNA and linear dsDNA.

**Conclusions:**

The results of this study demonstrated that the interaction between VP1 and DNA molecules exhibited various binding preferences that were dependent on the structural conformation of DNA. Taken together, the results of this report are the first to demonstrate that VP1 has no sequence-specific DNA binding activity. The particular binding preferences of VP1 might play multiple roles in DNA replication or encapsidation during the viral life cycle.

**Electronic supplementary material:**

The online version of this article (10.1186/s12917-018-1465-5) contains supplementary material, which is available to authorized users.

## Background

Chicken anaemia virus (CAV) is a common viral agent in chickens worldwide. CAV belongs to the genus *Gyrovirus* of the *Anelloviridae*, which have characteristics of circular single-stranded DNA viruses [1]. This virus frequently results in immunosuppression and anaemia in young chickens due to the destruction of T lymphoid tissue and aplasia of bone marrow, respectively, during virus infection [2–5]. Over 55% of the mortality rate and 80% of the morbidity rate were reported once the chicks were infected with CAV [6]. Therefore, determining how to prevent CAV infection in the poultry industry has becomes an important challenge. The CAV virion lacks an envelope around its capsid coat, and it shows significantly high resistance to environmental stress or chemical agents. Currently, an attenuated live vaccine is available and effective for immunization of chickens for controlling CAV infection. However, young chicks less than 2 weeks-old are susceptible to CAV infection when the live vaccine was used [5]. This consequence has led to the development of a subunit vaccine, including DNA or protein based vaccine, over the past decade.

CAV is a relatively small virus approximately 23 nm in diameter. A total of three open reading frames (ORFs) were involved in the viral genome and have a length of 2.3 kb [2]. These ORFs respectively encode a 51 kDa VP1 protein, a 28 kDa VP2 protein and a 13 kDa VP3 protein. VP2 has dual-specificity phosphatase activity. VP3 is also referred to as apoptin with apoptosis-inducing activity. VP1 is the sole structural protein, which is the major component responsible for capsid assembly [5, 7, 8]. Currently, VP2 and VP3 proteins have been the focus of investigations of virus pathogenicity [9, 10]. In addition to its importance in the viral life cycle, VP3 has also demonstrated apoptosis-inducing activity as well as medical applications for anti-cancer treatments for humans in many previous studies [11–14]. VP1 can interact with VP2 and then significantly elicit the production of virus-neutralizing antibodies in the host in terms of immunogenicity studies [15]. Therefore, VP1 is thought to be a good candidate for an immunogen to develop a subunit vaccine [15].

**Fig. 1:**
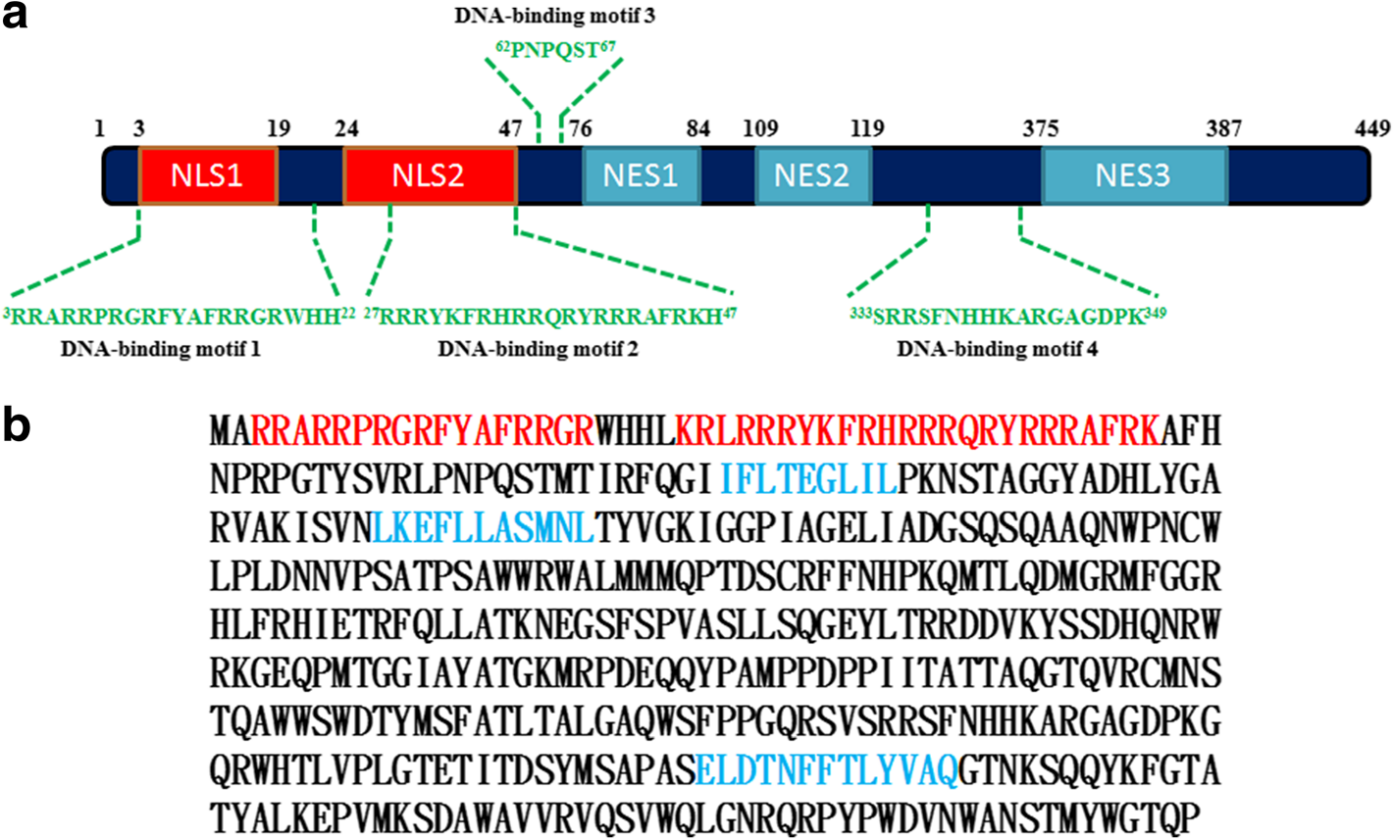
Prediction results for putative NLS, NES and DNA-binding motifs on CAV VP1 proteins. **a** Schematic diagram representing the distribution regions of putative functional motifs: nuclear localization signals (NLS), nuclear export signals (NES) and DNA-binding motifs on CAV VP1. Two NLS that separate at the N-terminus of VP1 were predicted by PSORT II software (http://psort.hgc.jp/form2.html) and three NES, which are mainly at the C-terminus of VP1 were predicted by NetNES 1.1 Server software (http://www.cbs.dtu.dk/services/NetNES/), respectively. The software DP-Bind (lcg.rit.albany.edu/dp-bind) was used for putative DNA-binding motif prediction, and the predicted amino acid sequences were described on the diagram. **b** Predicted amino acid sequence results of NLS and NES for CAV VP1 were indicated by red (NLS)- and green (NES)-labels, respectively

DNA replication of DNA viruses usually occurs in the nucleus of infected cells. Thus, to establish a productive infection, viral DNA with a high molecular weight needs to cross the nuclear envelope through protein-mediated nuclear transportation after infecting the cells [16]. Approximately 90% of karyophilic proteins containing nuclear localization signals (NLSs) are directed to the nucleus. The NLS sequences usually overlap with the DNA binding domains. Therefore, proteins for nuclear transport possess both DNA binding and NLS activities [17, 18]. CAV is first *Gyrovirus* to be discovered and isolated [3]. By cloning and sequencing the viral genome, previous studies have reported an N-terminal 40 amino acid sequence within the predicted amino acid sequence of VP1 that demonstrated a significant (46%) degree of similarity to the protamine protein in Japanese quails. This specific region within the N-terminus of VP1 contains high arginine content and might confer an ability to VP1 to bind and protect DNA [19]. Using online software, including PSORT II (http://psort.hgc.jp) and DP-Bind (lcg.rit.albany.edu/dp-bind/), the VP1 protein was analysed in this study. A total of four putative DNA-binding motifs and two putative NLSs were found and predicted within the CAV VP1, as illustrated in Fig. 1. A previous researcher reported that transient expression of GFP-VP1 in the plant cells has been observed throughout the nucleoplasm [20]. This outcome demonstrated that VP1 protein might be a nuclear protein. Other circular single-stranded DNA virus, such as duck circovirus (DuCV) and beak and feather disease virus (BFDV), have exhibited a pattern of N-terminal amino acid residues within the capsid protein that are highly basic amino acid rich sequences with nuclear localization signals and DNA binding activity [21, 22]. Based on these findings, N-terminal amino acid residues within the capsid protein of circovirus are very similar to the CAV of *Gyrovirus*. However, there is still a lack of direct evidence to prove and characterize the DNA binding ability or nuclear localization activity of VP1.

In this study, to gain insight into the role of the capsid protein VP1 in the life cycle of CAV, we have investigated the physical interactions of CAV VP1 with the viral DNA. A recombinant *E. coli* expression system was used to express the recombinant VP1 of CAV following our previous study [29]. The intracellular localization of the CAV VP1 was observed in MDCC-MSB1 cells or CHO-K1 cells using fluorescent green protein in the nucleoplasmic compartment. The DNA-binding activity of VP1 was also systemically examined. To the best of our knowledge, this is the first report to verify the DNA binding activity of the CAV capsid protein, VP1.

## Results

### Functional prediction of the CAV VP1 protein

Previous studies have shown that only the VP1 protein is located in the CAV virion. Thus, VP1 is also thought to be a DNA-binding protein that is responsible for the encapsidation of a viral genome during virus assembly. Presently, VP3 is the only one of three CAV viral proteins that has exhibited DNA binding activity in previous studies [23]. However, the function of VP1 on nucleic acid binding is still unknown. To gain insight into the role of the capsid protein VP1 in DNA binding, the bioinformatics software DP-Bind (http://lcg.rit.albany.edu/dp-bind/) was applied to analyse the features of DNA binding motifs within the amino acid sequence of the VP1 protein. Computational results of the DNA binding motif from the VP1 protein are shown in Fig. 1. Four potential DNA binding motifs were predicted by the DP-Bind program, and the putative motif position spanned from amino acids residues 3 to 22, 27 to 47, 62 to 67 and 333 to 349. According to these predicted results, VP1 might be having potential activity to bind DNA molecules. However, further investigation is still needed to verify the DNA binding activity of VP1.

### Expression and purification of the recombinant CAV viral protein, VP1 and VP3

**Fig. 2:**
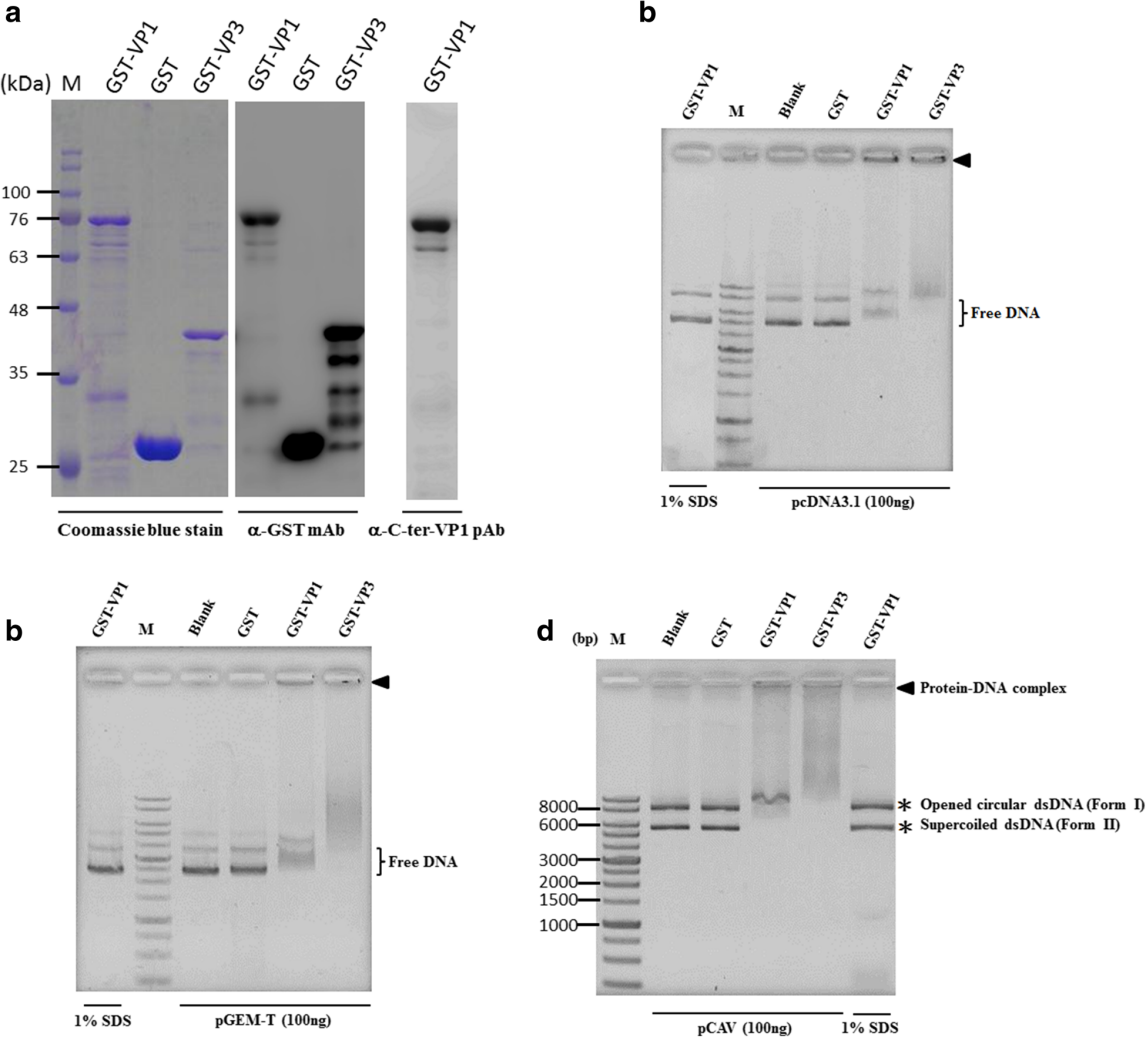
The VP1 protein has DNA-binding ability with no sequence specificity. The recombinant GST and GST-fused proteins were prepared by *E. coli* overexpression and purified through GST affinity chromatography. Purified results were analysed by SDS-PAGE with Coomassie blue staining and Western blotting with an anti-GST monoclonal antibody or anti-C-ter-VP1 polyclonal antibody (**a**). The purified proteins were used for DNA binding ability by an agarose gel shift assay with different DNA sequences of plasmid preparation of pcDNA3.1 (**b**), of the pGEM-T easy vector (**c**), and of pCAV containing the whole CAV genome (**d**). The binding activity of the VP1 protein was determined by comparing the existence of DNA fragments for the protein-DNA complex and DNA patterns from the blank (no-protein used), negative control (GST only) and positive control (GST-VP3). To confirm the observed DNA migration results that were induced by bound recombinant proteins, the protein-DNA experimental samples were mixed with 1% SDS as a protein denaturant (underline lane-labelled 1% SDS). Lane M, DNA ladder marker. Bold triangles indicated the protein-DNA complex formed by tested proteins and plasmids. Asterisks indicated the two conformations of plasmid DNA, including the relaxed form (Form I), and another was the supercoiled form (Form II)

To examine the DNA-binding activity of VP1, *E. coli* was used to express recombinant VP1 protein. Recombinant VP3 protein was also expressed as a positive control for the evaluation of DNA-binding activity. As shown in Fig. 2a, after purification by a GST affinity column, the purity and antigenicity of purified GST-fused VP1 and VP3 were determined by SDS-PAGE and Western blotting, respectively. This result confirmed the integrity of the two recombinant proteins.

### CAV VP1 is a nuclear protein that binds DNA with no sequence specificity

To elucidate whether the CAV VP1 protein is a DNA-binding protein, purified recombinant VP1 protein was added to circular dsDNA, pCAV, pcDNA3.1 and pGEM-T plasmids and incubated for 1 hour under 37 °C. After incubation, the occurrence of protein-DNA interaction was analysed by DNA movement on agarose gel. As illustrated in Fig. 2b, c and d, the migrations of pCAV, pcDNA3.1 and pGEM-T plasmid on the agarose gel were significantly reduced and shifted towards a pattern with a higher molecular weight. This result is very similar to the reduction in DNA migration that arose from binding VP3 to DNA (Fig. 2b, c and d). In contrast, no reduction in DNA migration occurred when the VP1 protein was absent or when GST protein was loaded with the addition of circular dsDNA plasmid. Moreover, when VP1 protein was pre-treated with 1% sodium dodecyl sulfate (SDS), the denatured VP1 no longer had DNA binding activity (Fig. 2b, c and d).

With respect to pCAV, which is a pcDNA3.1 plasmid carrying the entire CAV genome, the VP1 protein also displayed its DNA binding activity in terms of altered DNA migration pattern, as illustrated in Fig. 2b. In other words, regardless of whether the plasmid DNA used in the protein-DNA binding reaction was from pcDNA3.1 or pCAV, there was no significant effect on the resulting pattern of DNA migration (Fig. 2b, c and d). However, it is worth noting that the DNA migration of the circular dsDNA plasmid in the agarose gel displayed open circular dsDNA (form I, with a higher molecular weight pattern) and supercoiled dsDNA (form II, with a lower molecular weight pattern), simultaneously (Fig. 2b). The VP1 protein was bound to supercoiled dsDNA. which demonstrated that DNA shifting was more obvious than open circular dsDNA.

**Fig. 3:**
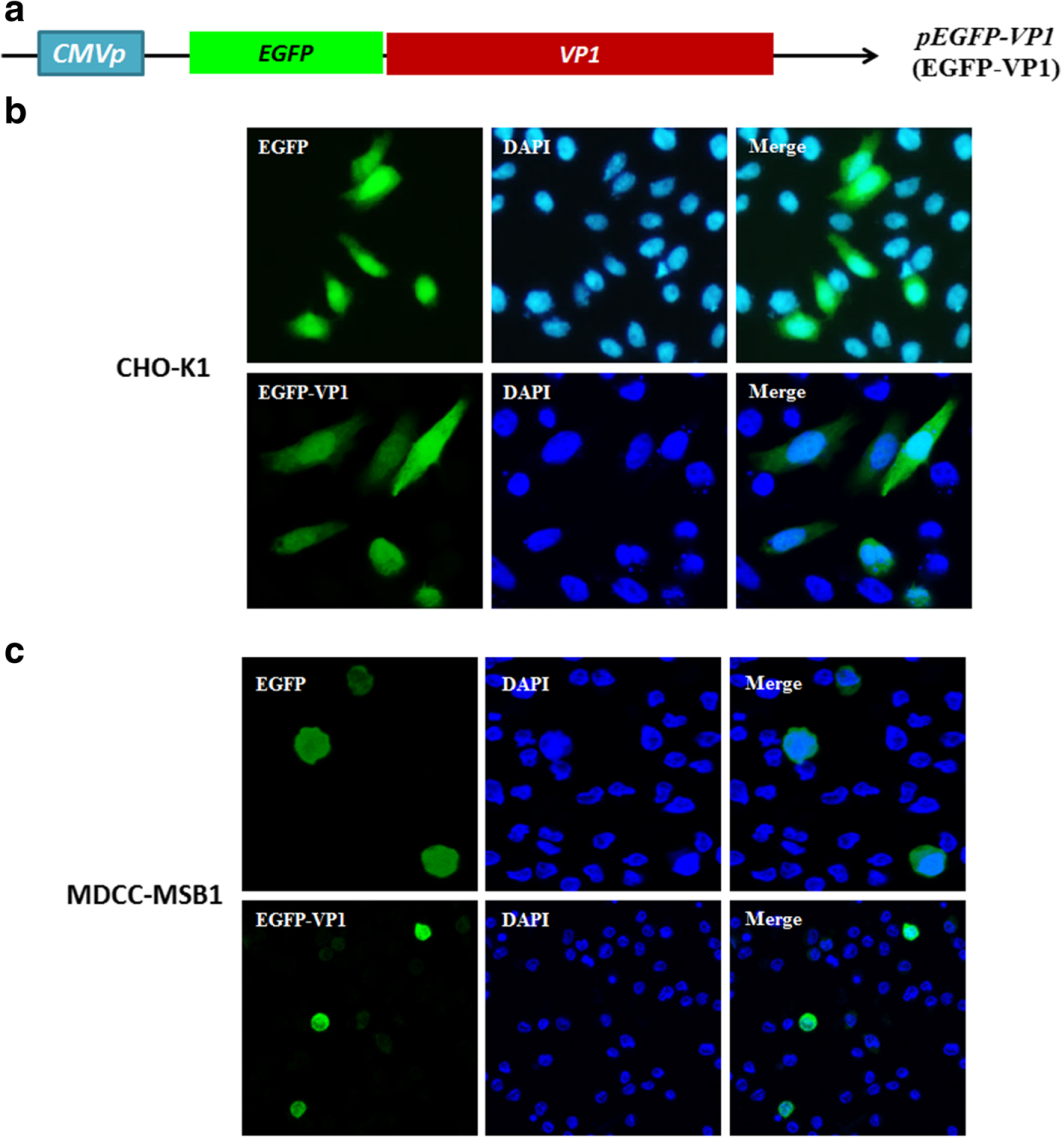
The nucleocytoplasmic distribution characterization of VP1 protein in CHO-K1 and MDCC-MSB1 cells. To realize the subcellular distribution of VP1, the full-length VP1 gene included the fused whole EGFP gene at the 5′-terminus to generate the EGFP-VP1 expressing plasmid pEGFP-VP1 as illustrated in a schematic diagram (**a**). After 48 h post-transfection with the above plasmid in CHO-K1 cells (**b**) and MDCC-MSB1 cells (**c**), both cell types were fixed and stained with DAPI to reveal the nuclei. The subcellular localization of VP1 was determined by green fluorescence detection through confocal fluorescence microscopy

Next, to confirm that VP1 not only has DNA binding activity but also has nuclear localization activity, we constructed a transit expression plasmid, pEGFP-VP1, which is a pcDNA3.1 vector carrying the VP1 gene fused to a GFP gene for cell transfection (Fig. 3a). When pEGFP-VP1 was respectively transfected into chicken lymphocytes, MDCC-MSB1 cells and Chinese Hamster Ovary (CHO) K1, the localization of GFP fluorescence was observed using confocal microscopy (Fig. 3b and c). As illustrated in Fig. 3b and c, GFP-VP1 and DAPI staining coincided significantly in the nuclei of MDCC-MSB1 cells (Fig. 3c). Additionally, GFP-VP1 was partially distributed and displayed in the cytoplasm of MDCC-MSB1 cells (Fig. 3c). A similar pattern of the distribution of GFP-VP1 was also presented in the CHO-K1 cells (Fig. 3b). These results clearly demonstrated VP1 is also a nuclear protein and distributed within the nucleocytoplasmic compartment. Taken together, these results indicated that CAV VP1 is a DNA-binding protein with nuclear localization activity, and its DNA binding is not specific to a particular sequence.

### CAV VP1 binds DNA with a conformational preference

**Fig. 4:**
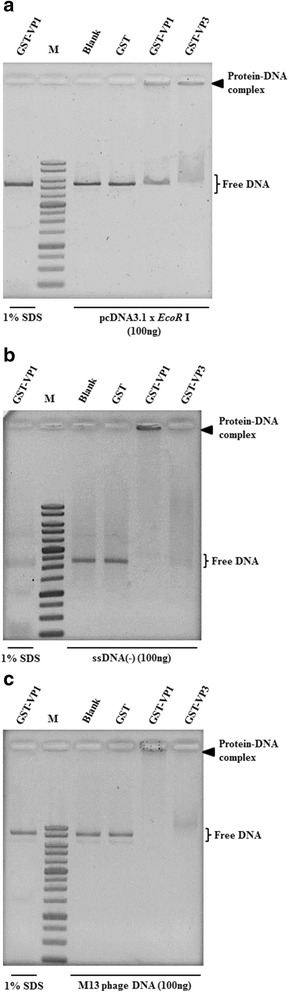
VP1 protein binds to various DNA molecules. Purified GST and GST-fused proteins were used for analysing the interaction of recombinant proteins with various DNA samples, such as linear dsDNA (**a**), minus-strand ssDNA (**b**), and M13mp18 phage DNA (**c**). All DNA samples were generated by different preparations as described in the Materials and Methods. After the agarose gel shift assay, the DNA fragment signals were observed by EtBr staining. The 1% SDS (underline lane-labelled 1% SDS) was also used to confirm the retardation caused by tested proteins. Lane M, DNA ladder marker. Bold triangles indicate the protein-DNA complex formed by the tested protein and DNA molecules. The “pcDNA3.1 x *Eco*R I” indicated generation of the linear form of pcDNA3.1 DNA digested by *Eco*R I

**Fig. 5:**
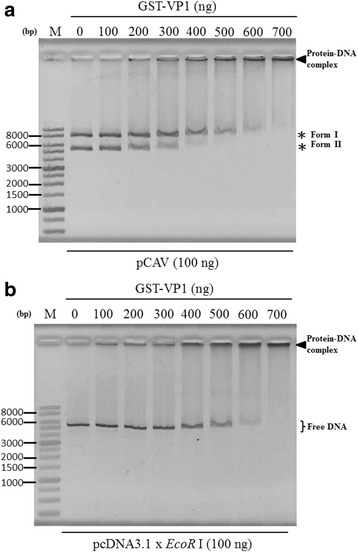
Dose-dependent analysis of VP1-dsDNA binding ability. Various concentrations of GST-VP1 were used to perform the dose-dependent analysis with a consistent concentration of plasmid pCAV (**a**) or linear dsDNA (**b**). The DNA fragment signals of the protein-DNA complex and differences in DNA migration patterns were more significant as the protein amount increased. Lane M, DNA ladder marker. Bold triangles indicate the protein-DNA complex formed by the tested protein and plasmids. Asterisks indicate the two conformations of plasmid DNAs, including the relaxed form (Form I) and a supercoiled form (Form II). The "pcDNA3.1x*Eco*RI" indicated generation of the linear form of pcDNA3.1 DNA digested by *Eco*RI

**Fig. 6:**
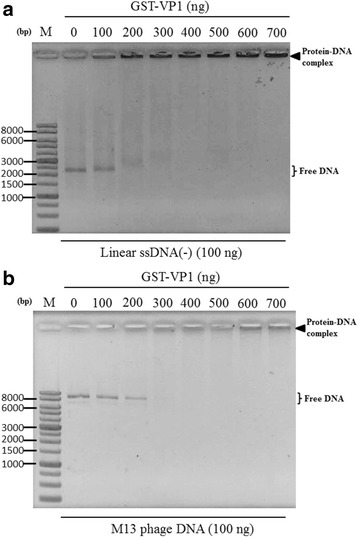
Dose-dependent analysis of VP1-ssDNA binding ability. Increased concentrations of GST-VP1 were incubated with consistent concentrations of minus-strand ssDNA (**a**) or circular ssDNA (**b**) to perform dose-dependent analysis. The disappearance of free DNA signals was more obvious when the protein amount increased. Lane M, DNA ladder marker. Bold triangles indicate the protein-DNA complex formed by tested proteins and DNA molecules

According to the results in Fig. 2b, the supercoiled dsDNA seems to interact with the VP1 protein more than opened dsDNA. Thus, to further address whether the DNA-binding activity of VP1 is affected by DNA conformation, various species of DNA molecules, such as linear dsDNA, circular ssDNA and linear ssDNA were used to confirm VP1 DNA binding activity. Because the DNA binding activity of VP1 has no sequence specificity as illustrated in Fig. 2b, c and d, the commercial circular single-stranded genome of the M13 phage was used as sample DNA instead of the real circular ssDNA genome of CAV. As illustrated in Fig. 4 with respect to all DNA species, DNA retardation occurred when the recombinant VP1 proteins were added to DNA molecules, such as linear pcDNA3.1 (linear dsDNA, Fig. 4a), the linear single strand of the CAV genome (minus and strand, Fig. 4b) and the genome of the M13 phage (circular ssDNA, Fig. 4c). Similarly, with respect to the VP3 protein, all kinds of DNA molecules were also bound by VP3 and reduced the migration of DNA. However, comparing the significance of DNA patterns between various protein-DNA complexes, different DNA molecules bound by VP1 protein demonstrated there were distinct migration patterns of DNA (Fig. 4). Therefore, to address the binding preferences of the VP1 protein to DNA molecules, various amounts of VP1 protein were added to equal amounts of different DNA molecules for analysis of protein-DNA interactions. By quantifying the amount of VP1 protein required for DNA binding with respect to pCAV (circular dsDNA), especially for its supercoiled form dsDNA, the results showed 200 μg of VP1 were required to initiate VP1 binding to DNA molecules (Fig. 5a, supercoiled, form II). At least 300 μg of VP1 were required for this interaction to occur between VP1 and opened dsDNA (Fig. 5a, opened, form I). Higher amounts of VP1 protein were used to bind circular dsDNA and reduced DNA migration patterns more significantly (Fig. 5a). Similarly, other DNA molecules, such as linear dsDNA (linearized pcDNA3.1, Fig. 5b), a linear single strand of the CAV genome (minus strand, Fig. 6a) and circular ssDNA (genome of the M13 phage, Fig. 6b), showed a similar pattern for protein-DNA interactions in the reaction mixture, with approximately 300 μg of VP1 required for linear dsDNA, 200 μg for the minus strand of linear ssDNA and 100 μg for circular ssDNA. In contrast, residual unbound DNA molecules representing the amount of DNA binding on the gel decreased if protein-DNA interaction occurred. Based on these results, the preferences in order of affinity to DNA with the VP1 protein in terms of the estimation of the percentage of unbound DNA molecules, which were sorted from low to high, were circular ssDNA (46.5% with respect to 300 μg of VP1), the linear minus strand of ssDNA (53.3% with respect to 300 μg of VP1), supercoiled circular dsDNA (82.6% with respect to 300 μg of VP1), linear dsDNA (84.7% with respect to 300 μg of VP1) and opened circular dsDNA (100% with respect to 300 μg of VP1). The comparison of binding preferences of VP1 protein to different conformations of DNA molecules are summarized in Table 1. More unbound DNA existed in the agarose gel, implying that this conformation of DNA exhibited a lower preference to interact with the VP1 protein. These results demonstrated that the interaction of VP1 with the DNA molecule exhibited various binding preferences that were dependent on the structural conformation of DNA.

**Table 1:** The ratio of unbound free DNA residue was determined by calculating the image intensity of free DNA fragments on an electrophoretic agarose gel after a VP1-DNA binding assay combining increasing amounts of recombinant GST-VP1 protein with certain DNA molecule conformations

	Concentrations of GST-VP1 protein (ng)				
	0	100	300	500	700
Open circular dsDNA (Form I)	100%	100%	100%	59.6%	11%
Supercoiled dsDNA (Form II)	100%	100%	82.6%	0%	0%
Linear dsDNA	100%	93.8%	84.7%	36.5%	0%
Linear ssDNA (−)	100%	92.9%	53.3%	0%	0%
M13 phage DNA	100%	90%	46.5%	0%	0%

The lower ratios indicate a higher preference of the VP1 protein for specific DNA conformations. The equation used in this study is presented under the table

## Discussion

DNA replication in the DNA viruses usually occurs in the nuclei of infected cells. Thus, viral DNA needs to cross the nuclear envelope through protein-mediated nuclear transportation to establish a productive infection after infecting the cells [16]. CAV is a non-enveloped, small DNA virus containing a circular ssDNA genome [2]. VP1, which is a major capsid protein of CAV, interacts with cells during virus infection, and the viral genome should theoretically be carried by VP1 to enter cells. The main question is what protein is conferred a function to direct a viral genome into the nucleus for sequential DNA replication? VP1 is thought to have a functional role to bind and direct the viral genome into the nucleus. However, there is still a lack of direct evidence to support this speculation. In our results from computational prediction, four putative DNA binding motifs were combined with the amino acid sequence of the CAV VP1 protein. Although the exact DNA binding motif was not determined in this study, it did not affect the characterization of VP1 DNA binding activity. After performing a DNA-binding assay to examine DNA migration, the VP1 protein of CAV was confirmed to have DNA-binding activity. Additional experiments are still needed for identifying major DNA binding motifs within VP1 protein. The confirmed DNA-binding activity in VP1 might be useful to further verify the underlying mechanisms of viral DNA replication. In addition, GFP-VP1 has demonstrated nucleo-cytoplasm shuttling activity (Fig. 3). The results imply VP1 is a nuclear protein to binds DNA molecules, such as those in the viral genome and travels into the nucleus during its early life cycle. In fact, we have not only predicted the presence of nuclear localization signals (NLSs) with PSORT II but also predicted nuclear exporting signals (NESs) with NetNES server (http://www.cbs.dtu.dk/services/NetNES/). These putative NLS motifs were within the amino acid sequence of VP1, which spanned from amino acid residues 3 to 19 (NLS1) and 24 to 47 (NLS2). For NESs, the putative motifs within the VP1 protein spanned from amino acid residues 76 to 84 (NES1), 109 to 119 (NES2) and 375 to 387 (NES3) (Fig. 1a). In terms of the observation of fluorescent of GFP-VP1, this result was confirmed according to computational predictions. In the early life cycle of the virus, viral DNA replication is an important stage for the establishment of productive infection [16]. Previous studies have reported circular, negative ssDNA of the CAV genome might be replicated through rolling-circle amplification [24]. During this stage, previous researchers isolated the viral replicative form (RF) DNA, which is an open circular dsDNA obtained from MDCC-MSB1 cells after being infected with the virus for 30 h [19]. In addition to the presence of closed and open circular dsDNA, circular ssDNA with genome-sized and small linear dsDNA of 800 bp were observed in the later stages of DNA replication [25]. In this study, all similar DNA molecules with different conformations, including linear ssDNA, which is derived from circular ssDNA, were used to examine the possible functional roles of the VP1 protein in DNA-binding activities. Additionally, other putative DNA binding motifs, especially for the initiation of rolling-circle amplification (RCR) within the VP1 protein, had also been reported and predicted in a previous study [26]. Three putative motifs were proposed that spanned from amino acid 313 to 320 (FATLTALG), 350 to 358 (GQRWHTLVP), and 399 to 408 (TATYALKEPV). These motifs might be interaction sites, such as the origin (Ori) site of the CAV genome, for interacting with VP1 for regulating DNA replication [26].

Generally, the DNA binding protein of most DNA viruses showed DNA binding activity with no sequence specificity. In this study, VP1 showed DNA binding characteristics with no sequence specificity similar to other DNA viruses, such as human papillomavirus and polyomavirus [27, 28]. Therefore, some other strategy of VP1 binding to the CAV viral genome should be adopted by the virus. With respect to the binding preferences of the VP1 protein to different DNA molecule conformations, VP1 was found to interact with circular ssDNA and exhibit a higher preference for this conformation, as shown in Table 1. This outcome might truly reflect the conditions of viral encapsidation for coating circular ssDNA of the CAV genome. Actually, circular ssDNA is prone to forming secondary structures with a high probability. This possibility was examined and confirmed with a computational prediction from the Mfold program (http://unafold.rna.albany.edu/?q=mfold/DNA-Folding-Form) (Additional file 1: Figure S1A, B). Similarly, linear ssDNA also has a high probability for forming secondary DNA structures (Additional file 1: Figure S2A, B). Thus, the binding preference of VP1 to linear ssDNA is surpassed only by the preference for circular ssDNA (Table 1). This difference truly meets our expectations. In fact, the sequence of linear ssDNA(−) was complemented with linear ssDNA(+). Then, linear ssDNA(+) was found to have significant VP1-DNA interaction in terms of the results of the DNA migration assay. The binding preference of VP1 to the linear plus-strand of ssDNA(+) is slightly lower than the linear plus-strand of ssDNA(−) (53.1% for 300 μg of VP1) (Additional file 1: Figure S3A, B). Other DNA molecules, such as supercoiled dsDNA, linearized ds DNA and opened dsDNA, displayed lower binding preferences to VP1, which might be a mechanism for VP1 protein to competitively bind various DNA molecules during different stages of the life cycle.

Taken together, this report is the first to show that VP1 has no sequence specificity for its DNA-binding activity and that its particular binding preferences might play multiple roles in DNA replication or encapsidation during a viral life cycle.

## Conclusion

In summary, the characterization of DNA binding activity of the VP1 protein was investigated in this study. VP1 was demonstrated to show DNA binding characteristics with no sequence specificity. In addition, the DNA binding activities of VP1 exhibited a differential preference to interact with various DNA molecules with different conformations. This information could be helpful for determining the biological roles of VP1 in the CAV viral life cycle.

## Methods

### Cell cultures, bacterial strains and plasmids

For Chinese Hamster Ovary (CHO-K1) cells, cells were purchased in 2014 from the Bioresource Collection and Research Center (BCRC 6006) in Taiwan. CHO-K1 cells were maintained in Ham’s F12 medium (HyClone, USA) supplemented with 10% FBS (HyClone, USA), 1% P/S (Penicillin/Streptomycin solution) (Gibco, USA). Chicken lymphoblast MDCC-MSB1 cells were purchased from the CLS Cell Lines Service GmbH in Germany in 2015. MDCC-MSB1 cells were grown in RPMI 1640 medium (HyClone, USA) supplemented with 10% FBS (HyClone, USA) and 1% P/S (Penicillin/Streptomycin solution) (Gibco, USA). All cells were cultured in appropriate tissue culture flasks and maintained in a cell culture incubator with 5% CO_2_ at 37 °C before experiments.

All expression constructs used in this study were maintained in the *E. coli* strain Top10F’ (Invitrogen, USA). The *E. coli* strain BL21 (DE3)-*pLys*S was transformed with protein expression plasmids and followed by IPTG induction to produce recombinant proteins as described in a previous study [29].

The construction of an expressed plasmid used for detecting subcellular localization was described below. The full-length of CAV VP1 was amplified by PCR using the specific primer sets wt-VP1-f: 5’-CCCGAATTCATGGCAAGACGAGCTCGC-3′, wt-VP1-r: 5’-CGCGTCGACTCAGGGCTGCGTCCCCCAGTA-3′ from the CAV VP1 template that was kindly provided by Dr. Yi-Yang Lien. The PCR product was then cloned into expression vector pEGFP-C2 (#6083–1, Clontech, USA) between *EcoR* I and *Sal* I sites to generate a recombinant plasmid named pEGFP-VP1.

### Expression and purification of the CAV VP1 and VP3 proteins

To purify the recombinant CAV VP1 and VP3 proteins, the previously created recombinant *E. coli* strains BL21 (DE3)-*pLys*S expressing VP1 and VP3 were used to express recombinant proteins [29]. The recombinant *E. coli* cells were cultured, and the harvested cells were disrupted and prepared following a previously described procedure [29]. Cells were spun down from 50 ml of culture supernatant and resuspended in GST resin binding buffer (140 mM NaCl, 2.7 mM KCl, 10 mM Na_2_HPO_4_, 1.8 mM KH_2_PO_4_, pH 7.3). After cell disruption, the resulting cell supernatant was loaded onto a GSTrap FF affinity column (GE healthcare, Piscataway, NJ) for protein purification following the operational conditions described in a previous study [29]. The total protein concentration of recombinant CAV VP1 and VP3 proteins was determined using a Micro BCA kit (Pierce, Rockford, IL) with bovine serum albumin as the reference protein. Purified VP1 and VP3 proteins were dialyzed against DNA-binding buffer (50 mM Tris-HCl, pH 7.5, 120 mM KCl, 1.0 mM EDTA, 0.5 mM DTT, and 30 mg/ml BSA) and analysed by sodium dodecyl sulfate-polyacrylamide gel electrophoresis (SDS-PAGE) and Western blotting. Purified proteins were stored at − 20 °C until required.

### Generation of nucleic acids used for the DNA-binding assay

Different DNA species, including circular dsDNA, linear dsDNA and circular ssDNA were used for assessing DNA-binding activity. The circular dsDNA, pcDNA3.1 (#V80020, Invitrogen, USA), pGEM-T easy vector (#A1360, Promega, USA) and pCAV were used for the DNA binding assay. The pCAV plasmid DNA was composed of a full-length Australian CAV strain CAU269/7 (GenBank #AF227982). The linear dsDNA was prepared from a pcDNA3.1 and pGEM-T plasmid by the cutting restriction enzyme *Eco*RI. Pure M13mp18 single-stranded DNA along with circular ssDNA materials were purchased from New England BioLabs (#N4040S, NEB, USA). All DNA molecules were diluted to 50 ng/ml with DNA-binding buffer and store at − 20 °C until required.

### Preparation of linear single-stranded DNA

The linear ssDNA was also used for the DNA binding assay. The preparation of linear ssDNA followed the protocol described in Marimuthu et al. [30] using the biotin-streptavidin separation method. First, a biotinylated DNA fragment containing the whole CAV genome was amplified by PCR using the EmeraldAmp Max PCR Master kit (Takara, Japan) from pCAV with a designed primer set, including the reverse biotinylated primer Biotin-CAV-r: biotin-labelled-GATTGT GCGGTGAACGAATTAG, and the forward regular primer CAV-f: GAATTCCGAGTGGTTACTATTC. After PCR amplification, the biotinylated PCR product was then immobilized on 40 μl Dynabeads M-280 Streptavidin magnetic beads (Invitrogen, USA) and incubated at 4 °C overnight. After washing the DNA-bonded beads twice with B/W buffer (5 mM Tris-HCl, pH 7.5, 0.5 mM EDTA, 1 M NaCl), the washed beads were incubated in 150 μl elution buffer (0.1 M NaOH, 1 mM EDTA, pH 13.0) to perform alkaline denaturation. Under the high alkaline environment, the desired non-biotinylated strand can be separated from the biotinylated strand and suspended in the supernatant. After magnet adsorption, the supernatants were collected, and the linear ssDNA was further purified by a PCR clean-up kit (Geneaid, Taiwan). The linear ssDNA was diluted to 50 ng/ml with DNA-binding buffer and store at − 20 °C until required.

### DNA binding assay

Purified GST, GST-VP1 and GST-VP3 proteins were diluted to 500 ng/μl with DNA-binding buffer and 500 ng of proteins were mixed with 100 ng of each DNA variant in a total of 20 μl of DNA-binding buffer. Then, each mixture was incubated for 30 min under 37 °C. The resulting sample was subjected to electrophoresis using a 0.8% agarose gel in a TAE buffer and, then the DNA was stained with ethidium bromide for the analysis of DNA migration.

### Cell transfection of CHO-K1 and MDCC-MSB1 cells

CHO-K1 cells were transfected by X-tremeGene HP DNA transfection reagent (Sigma, USA) according the manual’s protocol with a mixture containing 2 μg of plasmid pEGFP-VP1 and 4 μl of transfection reagent in 2.5 ml serum-free Opti-MEM medium (Gibco, USA). After incubating the mixture for 20 min at room temperature, the mixture was added drop by drop into cultured CHO-K1 cells in a 6-well plate. The 24 to 48 h post-transfection, the transfection effect was checked with a confocal fluorescent microscope.

For the transfection of MDCC-MSB1 cells, the 4 × 10^6^ log-phase grown MDCC-MSB1 cells were gently pipetted with 15 μg of plasmid pEGFP-VP1 first in serum-free RPMI 1640 medium and then the mixture was transferred into a 0.4-cm gap electroporation cuvette and the cuvette was harvested on ice for 5 min. The electroporation of MDCC-MSB1 cells was performed with a Gene Pulser II (Bio-Rad, USA) with a Time Constant Protocol set at 34 ms and an operating voltage of 300 V. After electroporation, the transfected cells were then cultured into complete medium in a 6-well plate for 24 to 48 h. Post-transfection, the expression of recombinant EGFP-VP1 proteins were analysed by confocal fluorescence microscopy to make sure the transfection was effective.

### Sample preparation for confocal microscopy observation

After transfecting CHO-K1 cells and MDCC-MSB1 cells with plasmid pEGFP-C2 or pEGFP-VP1, the fluorescent images were captured by a confocal fluorescence microscope in terms of the observation of protein fluorescent to verify the EGFP-expressed cells and EGFP-VP1-expressed cells. Transfected cells were collected and fixed with 4% formaldehyde in the dark. After washing the fixed cells twice to remove residual formaldehyde, the cells were stained in 0.1% PBS-T with 1 μg/ml DAPI for 5 min at 37 °C in the dark. Then, the stained cells were mounted with gelvatol medium (Sigma, USA) on a glass slide for confocal observation. Confocal laser scanning microscope (CLSM) images were captured from a Leica TCS SP8 confocal microscope and the images were integrated with LAS X Leica Confocal Software. EGFP fluorescence was observed through excitation at 488 nm and DAPI emitted blue fluorescence upon binding to DNA that was observed through excitation by UV light.

### Comparing the DNA conformational preference of VP1 protein through DNA analysis

To obtain the ratio of unbound DNA residues, a dose-dependent DNA-binding experiment was performed by combining various concentrations (0 to 700 ng) of GST-VP1 proteins with consistent amounts of different DNA variants. Next, the signal intensities of free DNA from DNA migration images after DNA-binding experiments were obtained using ImageJ software observation. The equation used in this study is presented below.

$$ \mathrm{Unbound}\ \mathrm{DNA}\ \mathrm{residue}\ \left(\%\right)=\frac{\mathrm{Image}\ \mathrm{intensity}\kern0.17em \mathrm{of}\kern0.17em \mathrm{free}\;\mathrm{DNA}\;\left(\mathrm{certain}\kern0.17em \mathrm{protein}\kern0.17em \mathrm{concentration}\right)}{\mathrm{Reference}\kern0.17em \mathrm{image}\kern0.17em \mathrm{intensity}\;\left(\mathrm{no}\;\mathrm{protein}\right)}\times 100\% $$

After dividing the signal intensity at a certain concentration of GST-VP1 proteins by the signal intensity in the absence of proteins (blank), the calculated ratio of unbound DNA residue was obtained to determine the DNA conformational preference for the VP1 protein.

## Additional file

Additional file 1: **Figure S1.** The putative secondary structure of the circular CAV genome as predicted by the software Mfold. **Figure S2.** The putative secondary structure of linear CAV genome. **Figure S3.** VP1 binds to linear plus-strand ssDNA. Purified GST and GST-fused recombinant proteins were used to analyse the interaction with linear plus-strand ssDNA in an agarose gel shift assay. (DOCX 1965 kb)
