# Supplementary material for: Characterization of the DNA binding activity of structural protein VP1 from chicken anaemia virus
Source: BMC Vet Res. 2018 May 4;14:155. doi: 10.1186/s12917-018-1465-5 (PMC5936033; doi:10.1186/s12917-018-1465-5)
Supplement: Supplementary file 1 — Figure S1. The putative secondary structure of the circular CAV genome as predicted by the software Mfold. Figure S2. The putative secondary structure of linear CAV genome. Figure S3. VP1 binds to linear plus-strand ssDNA. Purified GST and GST-fused recombinant proteins were used to analyse the interaction with linear plus-strand ssDNA in an agarose gel shift assay. (DOCX 1965 kb) [file 12917_2018_1465_MOESM1_ESM.docx]

**Supplementary data**

**Characterization of the DNA Binding Activity of Structural Protein VP1 from Chicken Anaemia Virus**

Guan-Hua Lai^1φ^, Ming-Kuem Lin^2φ^, Yi-Yang Lien^3^, Jai-Hong Cheng^4^, Fang-Chun Sun^5^, Meng-Shiunn Lee^6^, Hsi-Jien Chen^7^, Meng-Shiou Lee^2^*

^1^Graduate Institute of Biotechnology, National Chung Hsing University, Taichung 40402, Taiwan.

^2^ Department of Chinese Pharmaceutical Science and Chinese Medicine Resources, China Medical University, Taichung, Taiwan.

^3^ Department of Veterinary Medicine, National Pingtung University of Science and Technology, Pingtung, Taiwan.

^4^Center for Shockwave Medicine and Tissue Engineering, Department of Medical Research, Kaohsiung Chang Gung Memorial Hospital and Chang Gung University College of Medicine, Kaohsiung, Taiwan.

^5^ Department of Bioresources, Da-Yeh University, Changhua, Taiwan.

^6^ Research Assistance Center, Show Chwan Memorial Hospital, Changhua, Taiwan.

^7^ Department of Safety, Health and Environmental Engineering, Ming Chi University of Technology, New Taipei, Taiwan.

^φ^contributed equally to first author

*Corresponding author:

Department of Chinese Pharmaceutical Science and Chinese Medicine Resources, China Medical University, 91, Hsueh-Shih Road, Taichung, Taiwan.

Fax: +886-4-24075683

Tel.: +886-4-2205-3366 ext 5208

E-mail address: leemengshiou@mail.cmu.edu.tw (Lee, M-S.)

**Materials and Methods**

**Preparation of linear plus-strand ssDNA**

The linear plus-strand ssDNA was also prepared using the biotin-streptavidin separation method as described in the main manuscript. Primer set: forward regular primer CAV-r: GATTGTGCGGTGAACGAATTAG, and the reverse biotinylated primer Biotin-CAV-f: biotin-labelled-GAATTCCGAGTGGTTACTATTC. The biotinylated DNA fragment was amplified by PCR from the pCAV template. Next, the amplified biotinylated PCR product was mixed with 40 μl Dynabeads M-280 Streptavidin magnetic beads (Invitrogen, USA) and incubated at 4°C overnight for DNA-bead immobilization. After washing the immobilized beads twice with B/W buffer and then eluting the non-biotinylated strand by the incubating beads in 150 μl Elution buffer with alkaline denaturation, the linear positive-strand ssDNA was collected and purified with a PCR clean-up kit (Geneaid, Taiwan). The purified linear ssDNA product was diluted to 50 ng/ml with DNA-binding buffer and stored at -20°C until required.

**Prediction of the secondary linear structure of the circular CAV genome**

The putative secondary structures of the CAV genome were predicted by the online software Mfold (http://unafold.rna.albany.edu/?q=mfold/DNA-Folding_Form) using minus-strand or plus-strand linear or circular CAV genomes as input subjects.

**
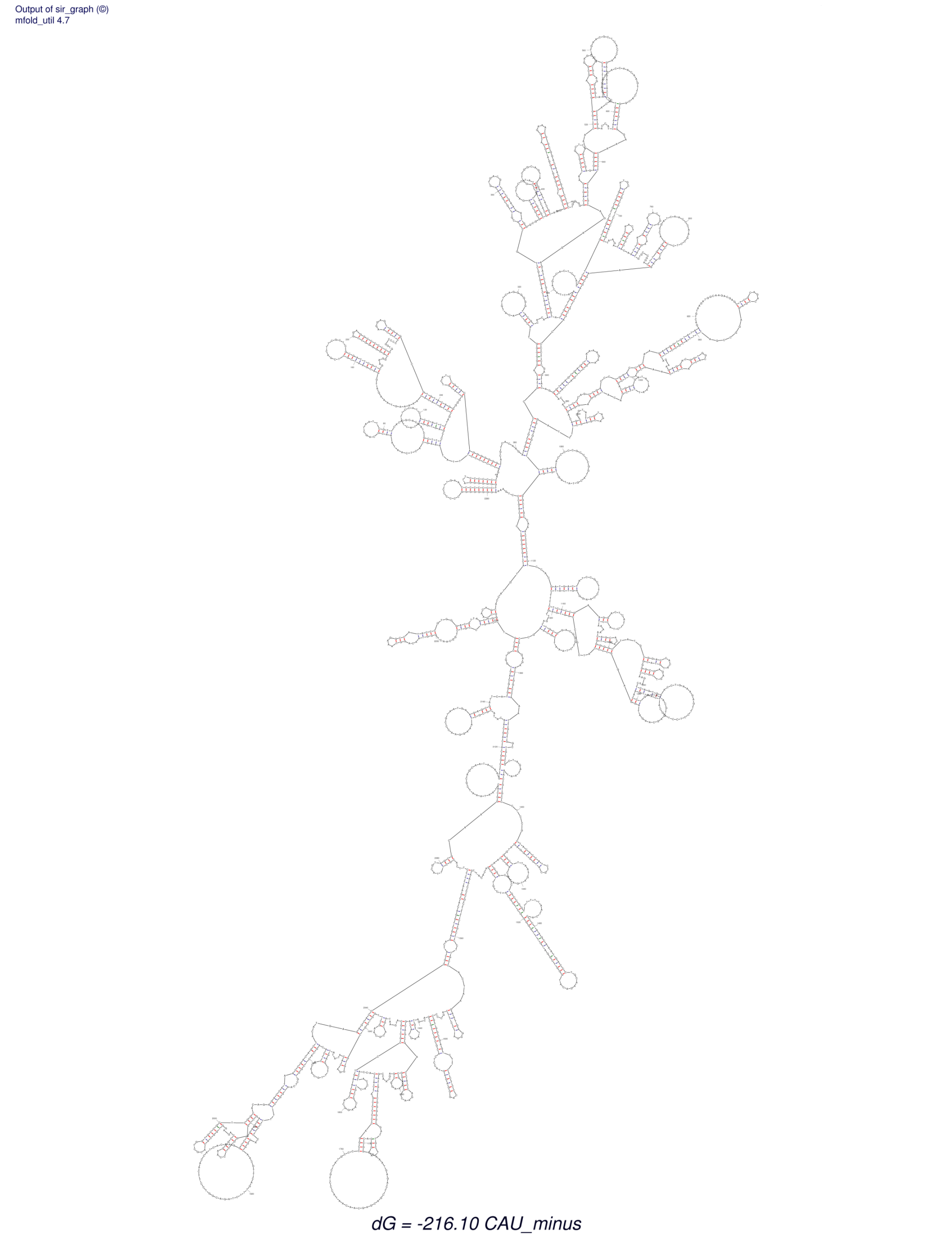
**

**A**

**Fig. S1**

**Fig. S1**

**
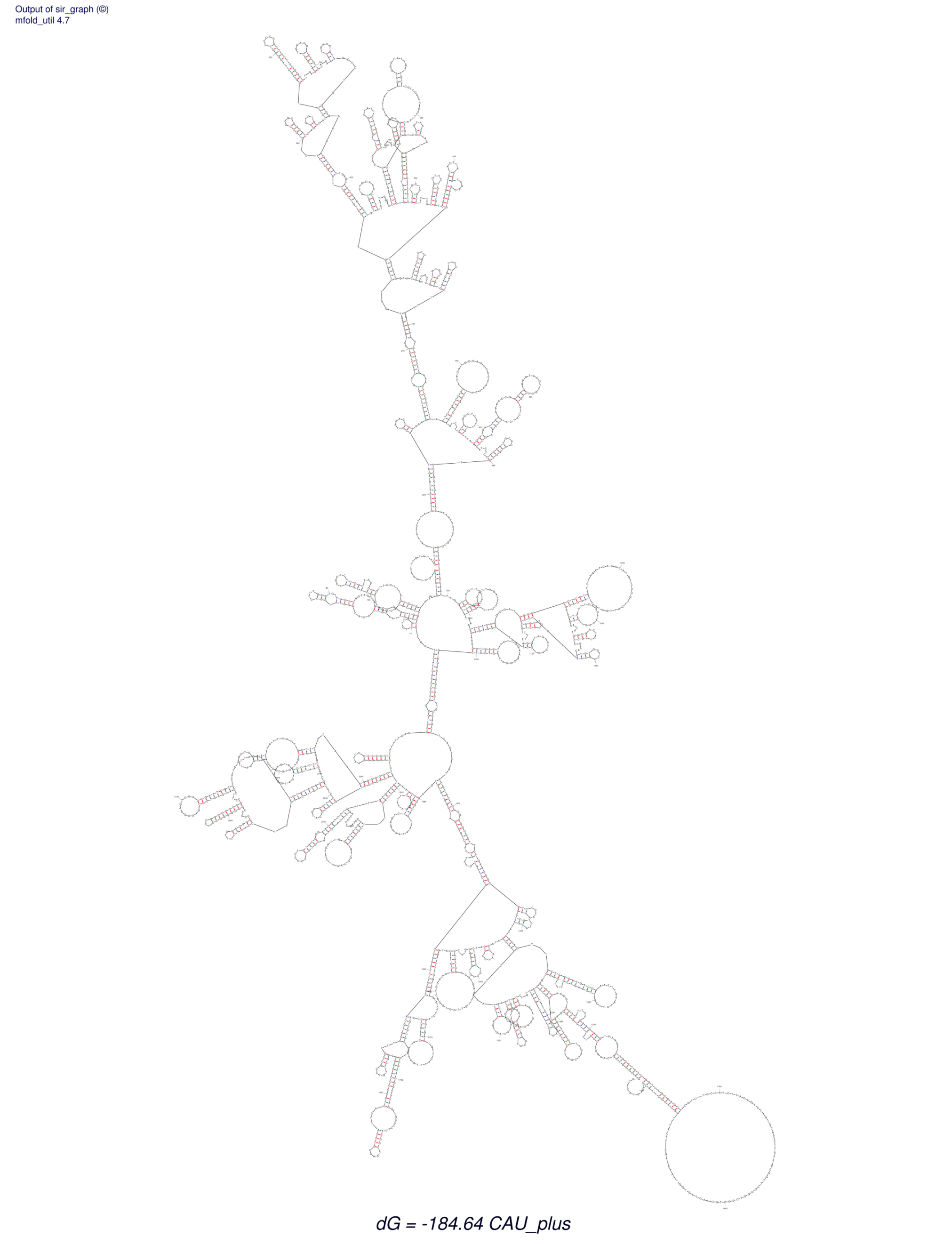
**

**B**

**Figure S1.** The putative secondary structure of the circular CAV genome as predicted by the software Mfold. The folded structure results were based on the DNA sequence of minus-strand (A) and plus-strand (B) CAV genomes.

**
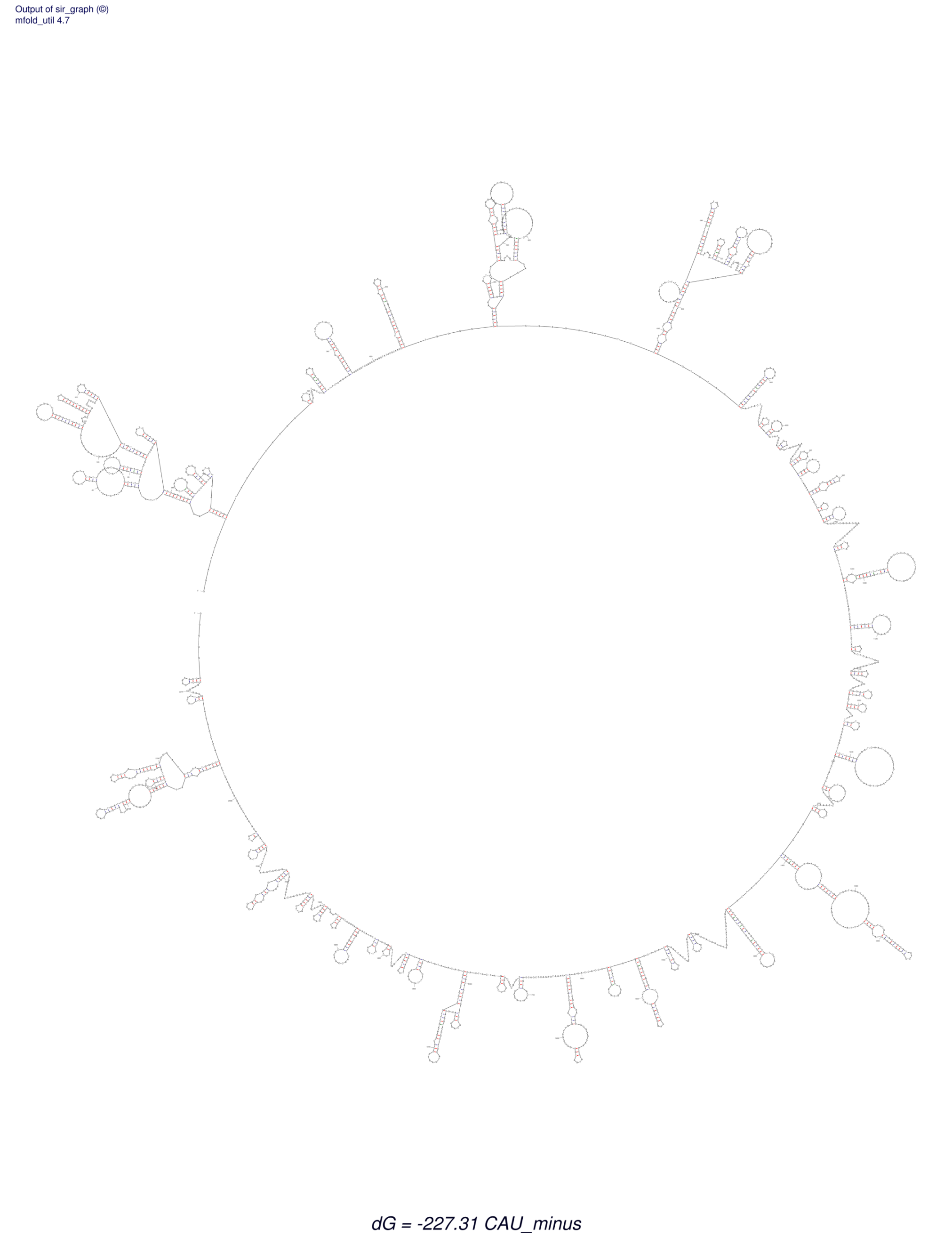
**

**Fig. S2**

**A**

**
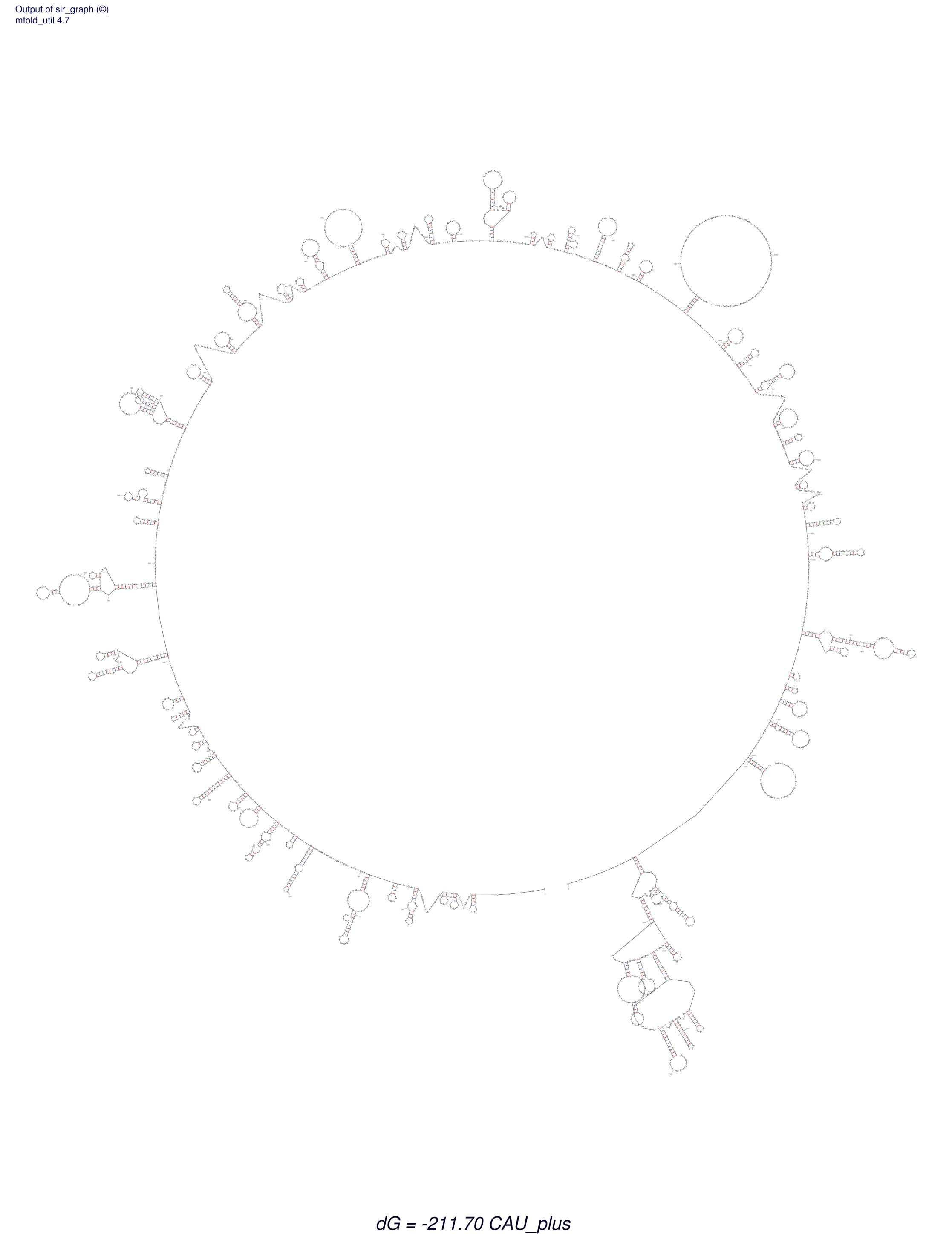
**

**Fig. S2**

**B**

**Figure S2.** The putative secondary structure of linear CAV genome. The DNA sequence of minus-strand (A) and plus-strand (B) CAV genomes were used in folded structure results predicted by Mfold software.

**Fig. S3**


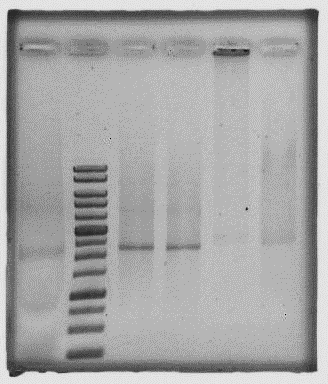


**M**

**Blank**

**GST**

**GST-VP1**

**GST-VP3**

**GST-VP1**

**ssDNA(+) (100ng)**

**1% SDS**

**Protein-DNA complex**

**A**

**Free DNA**


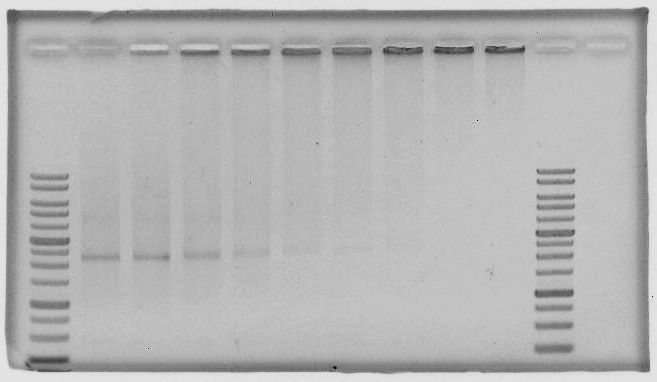


M

GST-VP1 (ng)

0

100

200

500

600

700

300

400

Linear ssDNA(+) (100 ng)

6000

1500

1000

8000

2000

3000

**(bp)**

**Protein-DNA complex**

**B**

**Free DNA**

**Figure S3.** VP1 binds to linear plus-strand ssDNA. Purified GST and GST-fused recombinant proteins were used to analyse the interaction with linear plus-strand ssDNA in an agarose gel shift assay (A). DNA fragments of the protein-DNA complex and DNA migration for the blank (no-protein used), negative control (GST only) and positive control (GST-VP3) were observed to determine the binding ability of VP1 to linear plus-strand ssDNA. The DNA migration results from bound recombinant proteins were confirmed by 1% SDS treatment (underline lane-labelled 1% SDS). Lane M, DNA ladder marker. Bold triangles indicate the protein-DNA complex formed by tested protein and ssDNA. The dose-dependent DNA-binding assays were performed using 100 ng linear plus-stranded ssDNA with increasing amounts (0-700 ng) of GST-VP1 protein and analysing the DNA migration in agarose electrophoresis (B). Lane M, DNA ladder marker. Bold triangles indicate the protein-DNA complex formed by tested protein and DNA molecules.
